# Cardiovascular responses to cognitive stress in patients with migraine and tension-type headache

**DOI:** 10.1186/1471-2377-7-23

**Published:** 2007-08-07

**Authors:** Rune B Leistad, Trond Sand, Kristian B Nilsen, Rolf H Westgaard, Lars Jacob Stovner

**Affiliations:** 1 Department of Neurosciences, Norwegian University of Science and Technology, Trondheim, Norway; 2 Department of Neurology and Clinical Neurophysiology, St. Olavs Hospital, Trondheim, Norway; 3 Department of Industrial Economics and Technology Management, Norwegian University of Science and Technology, Trondheim, Norway

## Abstract

**Background:**

The purpose of this study was to investigate the temporal relationship between autonomic changes and pain activation in migraine and tension-type headache induced by stress in a model relevant for everyday office-work.

**Methods:**

We measured pain, blood pressure (BP), heart rate (HR) and skin blood flow (BF) during and after controlled low-grade cognitive stress in 22 migraineurs during headache-free periods, 18 patients with tension-type headache (TTH) and 44 healthy controls. The stress lasted for one hour and was followed by 30 minutes of relaxation.

**Results:**

Cardiovascular responses to cognitive stress in migraine did not differ from those in control subjects. In TTH patients HR was maintained during stress, whereas it decreased for migraineurs and controls. A trend towards a delayed systolic BP response during stress was also observed in TTH. Finger BF recovery was delayed after stress and stress-induced pain was associated with less vasoconstriction in TTH during recovery.

**Conclusion:**

It is hypothesized that TTH patients have different stress adaptive mechanisms than controls and migraineurs, involving delayed cardiovascular adaptation and reduced pain control system inhibition.

## Background

Prolonged physiologic (e.g. autonomic) responses to a stressor, or insufficient recovery from stress, may cause disease, chronic pain or other subjective complaints [1-3]. Stress may also trigger headache in both migraine and tension-type headache (TTH) patients [4-7]. In other headache syndromes (e.g. "trigeminal autonomic cephalalgias") there seems to be a clear association between headache attacks and autonomic hyperactivity [8,9], and migraine symptoms in the prodromal phase and during attacks (e.g. nausea and vomiting) suggest autonomic imbalance also in these patients. Trigeminal and brainstem dysfunction is also implicated in migraine during attacks [10-12], and pain sensitivity is increased in TTH suggesting abnormal pain processing [13,14]. Because of the known interactions between autonomic and pain control centres in the brainstem (e.g. for the baroreceptor reflex [15]), and because autonomic hyperactivity may sensitize peripheral nociceptors [16], it makes sense to study if autonomic activation to stress is abnormal in migraine and TTH compared to healthy subjects, and if autonomic activation is related to the pain responses in these patients [17].

Cardiovascular responses to short-lasting acute stress have been measured in migraine but no clear pattern emerges [18-24], and data about responses and adaptation to more long-lasting cognitive stress are lacking. For TTH most studies concerning physiological responses to stressors have focused on muscular activity [25], and studies investigating cardiovascular responses to stressors report inconsistent findings [18,21,23,26-29]. Low-grade long-lasting cognitive stress may be more relevant to daily (e.g. work-related) stress than short-lasting stressors used in previously published studies, such as deep breathing tests, orthostatic tests, the cold pressor test and mental arithmetic tests. In addition, data about physiologic recovery after stress, which may be of particular importance as disease promoting factors [2,30], are insufficient in the headache research literature. Since migraine and TTH patients in some cases can have rather similar symptoms [31-33], although they are considered as clearly different syndromes, it was reasonable to include both entities in one study.

We have recently found that migraine and TTH patients have more stress-induced muscle pain and slower muscle pain recovery after long-lasting cognitive stress than controls [34,35]. This experimental task induces muscular activity and pain in the shoulders, neck and head of patients with migraine [34], TTH [34,36], cervicogenic headache [37], fibromyalgia [38,39] as well as in healthy controls [40]. However, muscular activation did not correlate with pain responses and no muscular response differences were found between migraine, TTH and controls [34]. Measuring cardiovascular and skin blood flow (BF) responses to stress and recovery after stress in parallel with pain in migraine and TTH may give insight into other potentially pain-inducing or contributing mechanisms in primary headache disorders.

The main questions were: do the early and the late autonomic activation pattern during stress and the recovery pattern differ in migraine, TTH and headache-free controls? Do the autonomic activation and recovery pattern correlate with increased pain during stress and recovery in migraine and TTH patients? We accordingly recorded blood pressure (BP), heart rate (HR) and skin BF development as well as head and shoulder/neck pain in these subjects during low-grade cognitive stress for one hour followed by 30 minutes of relaxation.

## Methods

### Subjects

Forty-four healthy control subjects, 35 women (mean age 39.7 years) and 9 men (36.6 years) and 40 patients with headache participated in this study. Twenty-two patients had migraine, 20 women (39.8 years) and 2 men (45.0 years), and 13 of these patients had aura. Eighteen patients had TTH, 9 women (33.8 years) and 9 men (35.7 years). Twelve of the TTH patients had chronic TTH. Detailed subject and headache history data are shown in Table 1. Patients were diagnosed after interview and physical examination by a neurologist according to the International Headache Society classification of headache from 1988 [41]. Control subjects did not suffer from headache or musculoskeletal pain for more than one day per month. Exclusion criteria were: neoplastic disease, hypertension, infectious disease, metabolic, endocrine or neuromuscular diseases, significant psychiatric disorders, connective tissue disorder, tendinitis, recent significant accident or injury, pregnancy, daily medication with neuroleptics, antiepileptics, Ca^2+^-blockers, β-blockers, antidepressants, and significant associated diseases affecting either the heart, lungs, cerebrovascular system, central or peripheral nervous system. Migraineurs with TTH more than 7 days per month were also excluded. The project was approved by the Regional Ethics Committee. All participants gave written informed consent and received NOK 500 (USD 75) for transport expenses and inconvenience. The participants were provided with written information concerning the aim of the study prior to the day of the stress test. The aim of studying pain and headache was mentioned, but the information focused on the practical details of the procedure.

**Table 1:** Background data on subjects included in the study. Pain/tension responses and recoveries are given in group means.

**Diagnostic group**	Controls (n = 44)	Migraine (n = 22)	Tension-type headache (n = 18)
Gender ratio (F:M)	35:9	20:2	9:9
Mean age (range)	39.0 (19–61)	40.2 (20–60)	34.7 (19–52)
Mean number of years with headache (range)	-	19.9 (7–37)	8.1 (0–32)
Number of subjects with chronic headache (%)	-	4 (18.2)	12 (66.7)
Mean duration (h) of headache attacks (range)*	-	29 (1–72)	34 (8-60)
Number of subjects with aura (%)	-	13 (59.1)	-
VAS pain response (range)	15.4 (0–66)	22.7 (0–54) ^2,3^	38.5 (3–88) ^1^
VAS pain recovery (range)	3.4 (0–47)	4.4 (0–19)^2,3^	16.4 (0–74) ^1^
VAS tension response (range)	21.2 (-13–82)	27.6 (-1–70)	32.7 (0–76) ^2^
VAS tension recovery (range)	13.0 (-11–75)	18.5 (-14–67)	26.4 (-16–65) ^1^

* One migraine patient had some attacks of short duration.

^1 ^Patients vs. controls, p ≤ 0.05. ^2 ^Patients vs. controls, 0.05 < p < 0.1. ^3 ^Migraine vs. TTH 0.05 < p < 0.1 (Mann-Whitney tests).

### Questionnaire and interview

A structured interview concerning headaches and musculoskeletal complaints (distribution, severity, and duration) was performed. One of these questions was: "Please state the level of general tension you have felt during the last 2–3 months", and the response was scored on a visual analogue scale (VAS) with endpoints: not tense – very tense. Participants also kept a headache diary for 7 days before and after the stress test. All subjects answered a questionnaire on marital status, weight, stimulant use, exercise habits, and sleep problems (data not shown). With the exception that migraineurs had lower alcohol consumption than controls (Chi-Square test, p = 0.034), there were no statistically significant differences in these parameters.

Thirteen of the 22 migraineurs reported a migraine attack within two days before the stress test, while twelve patients reported an attack within two days after the stress test.

### Physiological recordings

Muscular activity was recorded with surface electromyography (EMG) bilaterally in the trapezius, splenius, temporalis and frontalis muscles, as described in a previous paper [34]. Autonomic activity was measured indirectly by continuous recording of non-invasive finger BP (Portapres, TNO Biomedical Instrumentation, Amsterdam, The Netherlands) [42] and skin BF in the thumbs (Moorlab, time constant 0.02 s, low-pass filter 22 kHz; Moor Instruments Ltd, Devon, England). The BP cuffs were mounted on the intermediate phalanx on the left middle and ring fingers. Finger skin BF was measured bilaterally with the electrodes (fiber separation 0.5 mm) placed on the volar side of the distal phalanx (pulp) of the thumbs. The average from the left and right thumb was used for analysis, because a significant side difference was not found. Signals were sampled at 200 Hz. HR and BP was calculated with the Beatscope 1.0 software (TNO, Amsterdam, The Netherlands). Respiration was recorded with a thermistor (Embla S-AF-010, Flaga, Reykjavik, Iceland) below the nose with active elements in each nostril and in front of the mouth, but respiration frequency was not analysed in this study due to technical difficulties (Seven controls, eight migraineurs and two patients with TTH had corrupted respiration rate data).

### Procedure

**Figure 1:**
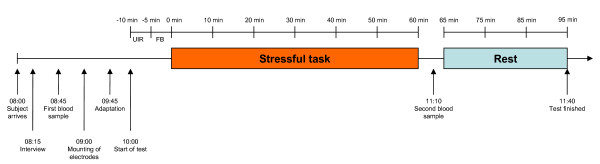
Overview of the test-day procedure. The subjects arrived at 08:00 and started with a structured interview, followed by the first blood sample. At approximately 09:00 the electrodes were mounted, and after a short adaptation period, the stress test started at 10:00. The stress test (incl. UIR and FB rest periods, stress period and recovery period) lasted for approximately 1 h 40 min.

The subjects were seated in an ordinary office chair without armrests and performed a two-choice reaction-time test presented on a PC monitor for 60 minutes [40]. The test involved a grid (7 columns by 5 rows) in which a large and a small square were placed randomly [43]. The subject was then presented with a suggestion on how to move the small square to superimpose it on the large square (for instance, "two up, four right"), and the subjects responded by pressing either "right" or "wrong" on a panel before them with their right index or ring fingers, respectively. Then the positions of the squares were changed, and a new suggestion was displayed. The subjects were instructed to carry out the assignment as fast and correctly as possible, and the computer provided feedback on performance by informing whether the answer was correct or not, and how fast the trial was performed (very slow, slow, normal, fast or very fast) [44]. The "normal" response for each subject was determined as the mean response time during a 5-minute trial period. The subjects were acclimated to the lab environment for 30 minutes, during which the procedure was explained and the recording equipment were mounted. The recording started with 5 minutes uninstructed rest (UIR) followed by 5 minutes active, instructed rest with visual EMG feedback (FB). FB-data are shown in figures but were not included in the statistical analysis because it was decided that UIR probably is a more realistic "real-life" baseline. The cognitive task was then performed for one hour (800–1500 trials), followed by 30 minutes recording during rest (recovery period). The subjects were asked to relax while seated and to move as little as possible during the recovery period. After the UIR and FB periods, at 10-minute intervals during the cognitive task, and at 10- minute intervals during the recovery period, the subjects were asked to mark on a VAS scale their level of pain (no pain – worst bearable pain), tension, fatigue and sleepiness. The different locations of pain corresponded with the positions of the EMG electrodes. Figure 1 shows an overview of the test day procedure. No patient had to be excluded because of headache attacks during the test. Venous blood was sampled before the test (immediately after the interview was concluded) and immediately after the stress period (after 60 minutes). Blood sample data will not be reported in this paper.

Some subjects had partly missing data due to technical difficulties: Two controls and two migraineurs had corrupted BP and HR data during the test and recovery period. One control was missing pain data at t_95min_, while one patient with TTH had corrupted BP, HR, BF, pain and tension data during the recovery period.

### Data analysis

Mean values for systolic blood pressure (SBP), diastolic blood pressure (DBP), HR and finger BF were calculated for the UIR and FB period, and for each 10-minute interval throughout the stress test and recovery period. These data were used in statistical ANOVA models (see below).

In order to minimize the number of correlations we also defined summery variables for autonomic response and recovery, and for pain response and pain recovery. Two summary variables were used for each autonomic variable (SBP, DBP, HR and finger BF) in correlation analyses: mean response (average of 60 minutes during stress – UIR) and mean recovery (average of 30 minutes recovery – UIR). The *pain response *was defined as the highest pain response (max pain at t_10–60min _– pain at t_0min_) among the eight location- and side-specific responses (trapezius, splenius, temporalis and frontalis muscles, left and right side). The muscle-specific pain data have been published in a previous paper [34]. The minimal pain during recovery was used first to calculate eight location- and side-specific pain recoveries (minimal pain at t_75–95min _– pain at t_0min_). Thereafter, the highest among these eight location- and side-specific pain recoveries was defined as *pain recovery*. These definitions were chosen because the highest (worst) pain during test (and the least complete recovery) was considered to most clinically relevant. Tension response and recovery were defined identically to the pain variables. Pain and tension variables are shown in Table 1.

### Statistical analysis

Baseline values were compared with univariate ANOVA (F_1 _models). Repeated measures ANOVA time × group interaction was used to explore differences in response patterns between groups. We do not report group-factor statistics in the present exploratory study because baseline values did not differ between groups (see results). Three different models with selected dependent variables were applied to explore different parts of the stress response and recovery curve. To examine how the novelty of the stressor influenced the subjects, the first 10 min and the baseline was compared in a F_2_-model (y = (baseline, 0–10 min)). This was described as the early (acute) stress response. After the first 10 min it was assumed that the novelty aspect of the stressor were gone, and we used a model named F_6 _with six repeated dependent variables (y = (0–10 min, 10–20 min, 20–30 min, 30–40 min, 40–50 min, 50–60 min)) to examine how the subjects adapted to the stressor. This was described as the late stress response. A F_3_-model with three dependent variables (y = (65–75 min, 75–85 min, 85–95 min)) was used to examine how fast and complete the subjects recovered from the stressor. The ANOVA models were corrected for non-sphericity by reduced degrees of freedom with Huyhn-Feldts method. Three-group ANOVA models were used as the primary analysis, followed by three two-group ANOVA models for the differences between controls and migraine, controls and TTH, and migraine and TTH respectively. Intra-group contrasts were explored by post-hoc Student's paired t-test. Group differences in pain and tension response and recovery (summary variables) were explored using Mann-Whitneys U-test. Univariate Spearman's rank order correlation analyses were used to explore associations between pain, tension and cardiovascular responses and recovery (summary variables). As our general statistical strategy involves a large number of comparisons, some might argue that there is a need for a multiple-comparison adjustment to control for type I errors [45]. We chose not to do this, as this would create other problems, such as an increase in type II errors [46,47]. Also, as the studies were considered to be mainly hypothesis-*generating *and not so much hypothesis-*controlling*, we believe that findings worthy of further research might be missed by applying too rigid criteria to the statistical analyses. A two-tailed significance level of <0.05 was considered significant in the statistical analyses. *P-*values within a range of 0.05–0.10 were defined as trends.

## Results

**Figure 2:**
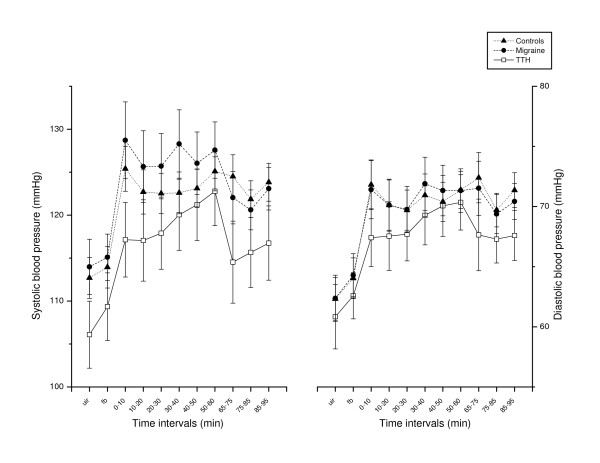
Systolic and diastolic BP development throughout the stress test and recovery period. Values are given as group means (SEM). UIR: Uninstructed rest period (baseline EMG). FB: EMG feedback aided rest period. 0 – 60: During the cognitive stress test. 65 – 95: Relaxation period after the test.

**Figure 3:**
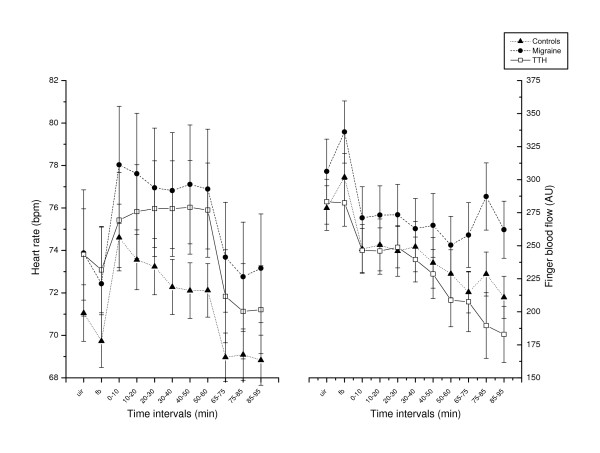
Heart rate and finger blood flow development throughout the stress test and recovery period. Values are given as group means (SEM). UIR: Uninstructed rest period (baseline EMG). FB: EMG feedback aided rest period. T = 0 – 60: During the cognitive stress test. T = 65 – 95: Relaxation period after the test.

There were no statistically significant differences between the three subject groups when comparing physiological baseline values (see F_1 _values in Table 2). Inspecting Figures 2 and 3, it appears that SBP, DBP and HR increased more abruptly and then decreased (i.e a "spiked" shape in Figures 2 and 3) at the start of the stressor in controls and migraineurs, but not in patients with TTH.

**Table 2:** Physiological mean values (SD) measured at baseline, during mental stress (0–60 min) and during the recovery period (65–95 min).

		Controls Mean (SD)	Migraine Mean (SD)	Tension-type headache Mean (SD)
**Systolic blood pressure (mmHg)**	Baseline	112.7 (15.4)	114.0 (14.4)	106.1 (16.5)
	0–10 min	125.4 (17.0)	128.7 (20.0)	117.1 (18.4)
F_1 _(2, 79) = 1.49, p = 0.23	10–20 min	122.7 (16.7)	125.7 (18.7)	117.1 (20.1)
F_6 _(7.0, 269.0) = 1.19, p = 0.31	20–30 min	122.5 (15.3)	125.7 (17.0)	117.9 (17.8)
F_2 _(2, 77) = 0.42, p = 0.66	30–40 min	122.6 (16.0)	128.3 (17.8)	120.0 (17.6)
F_3 _(3.3, 128.7) = 0.60, p = 0.63	40–50 min	123.1 (14.6)	126.0 (16.3)	121.2 (17.7)
	50–60 min	125.1 (14.9)	127.6 (14.7)	122.8 (17.0)
	65–75 min	124.5 (16.5)	122.0 (13.6)	114.5 (19.7)
	75–85 min	121.9 (14.0)	120.6 (10.4)	115.7 (16.8)
	85–95 min	123.8 (14.5)	123.1 (11.1)	116.8 (17.8)
**Diastolic blood pressure (mmHg)**	Baseline	62.3 (11.5)	62.4 (8.6)	60.8 (11.3)
	0–10 min	71.8 (13.4)	71.4 (10.8)	67.4 (10.2)
F_1 _(2, 79) = 0.13, p = 0.88	10–20 min	70.1 (14.0)	70.1 (9.3)	67.5 (12.1)
F_6 _(7.0, 268.9) = 0.77, p = 0.62	20–30 min	69.7 (11.0)	69.8 (8.6)	67.7 (9.4)
F_2 _(2, 77) = 0.77, p = 0.47	30–40 min	70.9 (11.6)	71.9 (9.9)	69.3 (10.4)
F_3 _(2.8, 107.9) = 0.34, p = 0.78	40–50 min	70.4 (10.6)	71.3 (9.4)	70.1 (10.7)
	50–60 min	71.4 (10.2)	71.3 (8.1)	70.3 (9.8)
	65–75 min	72.4 (13.7)	71.5 (10.1)	67.6 (12.3)
	75–85 min	69.7 (9.2)	69.4 (7.3)	67.3 (8.2)
	85–95 min	71.4 (9.5)	70.4 (6.7)	67.6 (8.5)
**Heart rate (beats/min)**	Baseline	71.1 (8.6)	73.9 (13.3)	73.8 (9.1)
	0–10 min	74.6 (10.3)	78.0 (12.4)	75.4 (9.5)
F_1 _(2, 79) = 0.76, p = 0.47	10–20 min	73.6 (9.2)	77.6 (12.7)	75.8 (9.4)
**F**_6_**(4.0, 156.9) = 2.87, p = 0.025**	20–30 min	73.2 (8.8)	76.9 (12.6)	76.0 (9.6)
F_2 _(2, 77) = 1.3, p = 0.28	30–40 min	72.3 (8.5)	76.8 (12.2)	76.0 (9.6)
F_3 _(3.9, 148.2) = 1.03, p = 0.39	40–50 min	72.1 (8.6)	77.1 (12.5)	76.0 (9.3)
	50–60 min	72.1 (8.3)	76.9 (12.6)	75.9 (9.4)
	65–75 min	69.0 (7.5)	73.7 (11.6)	71.8 (9.0)
	75–85 min	69.1 (7.9)	72.8 (11.5)	71.1 (9.2)
	85–95 min	68.8 (7.7)	73.2 (11.4)	71.2 (8.6)
Finger skin blood flow (AU*)	Baseline	278.5 (112.0)	306.2 (114.7)	283.3 (72.2)
	0–10 min	247.5 (121.9)	271.1 (110.4)	246.5 (70.5)
F_1 _(2, 81) = 0.52, p = 0.60	10–20 min	250.6 (130.3)	273.2 (103.6)	246.0 (74.3)
F_6 _(5.0, 189.8) = 049, p = 0.78	20–30 min	246.0 (126.8)	273.4 (108.0)	248.8 (66.2)
F_2 _(2, 81) = 0.07, p = 0.94	30–40 min	249.2 (125.8)	262.9 (107.4)	239.5 (72.1)
**F**_3_**(3.9, 157.0) = 2.3, p = 0.07**	40–50 min	237.1 (127.4)	265.4 (113.1)	228.6 (78.8)
	50–60 min	228.7 (120.4)	250.4 (102.3)	208.8 (85.3)
	65–75 min	214.9 (104.6)	258.1 (114.8)	207.6 (96.0)
	75–85 min	228.6 (110.6)	287.3 (119.1)	189.6 (105.4)
	85–95 min	211.0 (105.6)	262.1 (101.3)	182.9 (90.1)

F_1_: Oneway ANOVA F-statistic comparing baseline values between groups. F-statistic for group × time interaction in repeated measures ANOVA models is also tabulated for three different models in the left column: F_2_: Model for the *early response to stress*, with two intervals during the early stage of the stress test (baseline and 0–10 min). F_6_: Model for *adaptation or potentiation during ongoing long-lasting stress*, with six intervals during the stressful task (from 0–10 to 50–60 min). F_3_: Model to detect *fast versus slow recovery patterns *with three intervals during recovery (65–75, 75–85 and 85–95 min). p: Probabilities (degrees of freedom in parentheses) was adjusted for non-sphericity with Huyhn-Feldt's method. Significant interactions and trends in bold.

* AU: Arbitrary units.

### Cardiovascular responses to cognitive stress

ANOVA F_2 _analyses did not reveal any significant time × group interactions between the groups with regard to the initial (early) BP, HR or BF stress responses.

The late HR response pattern during ongoing stress from 0–10 to 50–60 min was significantly different between the three groups (see F_6 _time × group interaction value in Table 2) since HR adaptation in TTH differed significantly from HR adaptation in controls (Table 3). HR levels were stable in TTH patients whereas HR decreased after the initial response in controls (Figure 3).

**Table 3:** F-statistic for group × time interaction in two-group repeated measures ANOVA models.

	Controls vs Migraine	Controls vs TTH	Migraine vs TTH
**Systolic blood pressure**	F_2 _(1, 60) = 0.33, p = 0.57	F_2 _(1, 58) = 0.22, p = 0.64	F_2 _(1, 36) = 0.97,. p = 0.33
	F_6 _(3.3, 198.9) = 0.44, p = 0.74	F_6 _(3.5, 200.2) = 1.54, p = 0.20	F_6 _(3.5, 125.8) = 1.99, p = 0.11
	F_3 _(1.68, 102.3) = 0.24, p = 0.75	F_3 _(1.6, 89.7) = 1.06, p = 0.34	F_3 _(1.9, 67.4) = 0.47, p = 0.62
**Diastolic blood pressure**	F_2 _(1, 60) = 0.04, p = 0.85	F_2 _(1, 58) = 1.70, p = 0.20	F_2 _(1, 36) = 0.76, p = 0.39
	F_6 _(3.2, 190.9) = 0.22, p = 0.89	F_6 _(3.5, 202.7) = 1.27, p = 0.29	F_6 _(3.4, 121.7) = 0.82, p = 0.50
	F_3 _(1.4, 84.5) = 0.06, p = 0.88	F_3 _(1.3, 76.8) = 0.59, p = 0.50	F_3 _(1.6, 57.0) = 0.47, p = 0.59
**Heart rate**	F_2 _(1, 60) = 0.06, p = 0.80	F_2 _(1, 58) = 1.98, p = 0.17	F_2 _(1, 36) = 2.85, p = 0.10
	F_6 _(2.0, 122.2) = 1.46, p = 0.24	**F**_6_**(2.0, 115.8) = 5.06, p = 0.008**	F_6 _(2.1, 75.0) = 1.48, p = 0.23
	F_3 _(1.9, 117.0) = 1.83, p = 0.17	F_3 _(2.0, 113.4) = 0.83, p = 0.44	F_3 _(1.9, 65.1) = 0.12, p = 0.88
**Finger skin blood flow**	F_2 _(1, 64) = 0.06, p = 0.81	F_2 _(1, 60) = 0.15, p = 0.70	F_2 _(1, 38) = 0.01, p = 0.94
	F_6 _(2.5, 161.1) = 0.31, p = 0.79	F_6 _(2.2, 133.1) = 066, p = 0.53	F_6 _(2.7, 100.6) = 0.57, p = 0.61
	F_3 _(1.9, 124.3) = 0.66, p = 0.52	**F**_3_**(2.0, 119.5) = 3.21, p = 0.04**	**F**_3_**(1.9, 72.7) = 3.47, p = 0.04**

F_2_: Repeated measures ANOVA model with two intervals during the early stage of the stress test (baseline and 0–10 min). F_6_: Model with six intervals during the stressful task (0–60 min). F_3_: Model with three intervals during recovery (65–95 min). p: Probabilities (degrees of freedom in parentheses) was adjusted for non-sphericity with Huyhn-Feldt's method. Significant interactions in bold.

The SBP response tended to increase from the early (0–10 min) to the latest (50–60 min) part of stress (Student's paired t-test, p = 0.051) in TTH, while responses were stable in migraine and in controls (p > 0.66; Figure 2). SBP tended to decrease from 0–10 to 10–20 min in migraine patients (Student's paired t-test, p = 0.050) while no difference was found in TTH (p = 0.97). Significant ANOVA time × group differences were not found in SBP and DBP adaptation during the stress test however (F_6 _models in Table 2 and 3),

### Cardiovascular recovery after cognitive stress

TTH patients had a significant F_3 _time × group interaction for finger blood flow during the recovery period, compared to controls and migraine patients (Table 3). Figure 3 shows that finger blood flow in TTH patients continued to decrease throughout the recovery period, whereas this did not happen in the other groups.

### Relationship between pain, tension and cardiovascular responses and recovery

**Figure 4:**
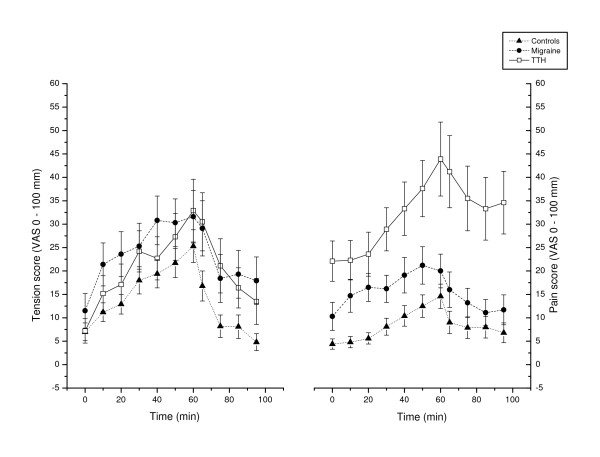
Tension and pain development throughout the stress test and recovery period. Values given as group means (SEM), where maximal reported pain (from the trapezius, splenius, temporalis and frontalis areas, irrespective of side) for each subject was used in the calculations. T = 0 – 60: During the cognitive stress test. T = 65 – 95: Relaxation period after the test.

In patients with TTH, mean finger BF recovery were related to the maximal pain response (r_s _= 0.49, p = 0.047), meaning that a high pain response was related to less finger BF reduction in recovery. There were no correlations between maximal pain responses and BP or HR responses, or between pain recovery and mean cardiovascular recovery, in any of the diagnostic groups. Pain responses were abnormally large while pain recovery were delayed in TTH patients compared to controls while perceived tension responses did not significantly differ between groups (Table 1, Figure 4). TTH patients also had significantly less recovery from tension compared to controls. There were no correlations between tension and cardiovascular responses and recovery for any of the three groups.

## Discussion

Controls, and to a certain degree also migraineurs, responded to the stressor in the present study with a rapid increase followed by a relatively fast decrease in BP and HR, giving the curve a spike-like shape. However, in TTH patients, the SBP, DBP and HR profiles increased slowly and did not decrease during the stress test. A trend towards a different SBP profile was found when comparing the first and last 10-min interval in controls and TTH. The possible lack of HR-adaptation during stress reflects the lack of a HR-spike (followed by a decrease in HR) in TTH. A reduced early cardiovascular response to mental stress, with the heart rate response inversely correlated to the pain response, was found for fibromyalgia patients in a study with a similar design [48]. Cardiac (HR) adaptation to mental stress has previously been reported in healthy students [49], while deficient cardiac adaptation to calculative mental stress has been found in migraine patients [50]. The migraine patients in our study did not show signs of deficient HR adaptation to stress. One may interpret the lack of an acute spike at the start of the cognitive task and the lack of HR adaptation as evidence of a deficient adaptive mechanism (or decreased autonomic excitability) to low-grade cognitive stress in TTH patients. It should be noted that due to a low sample size, especially in the TTH group, these results are tentative and are considered to be hypothesis-generating and not hypothesis-controlling.

HR in migraineurs recovered as much during the relaxation phase as controls. This is in accordance with another study [19] which did not show a difference in HR recovery between students with migraine and controls after three minutes of mental arithmetic, although the authors reported faster recovery in peripheral resistance in migraine compared to controls. On the other hand, Holm *et al*. [20] found that migraineurs had delayed HR recovery after four minutes of stressful speech-preparation. Methodological differences make it difficult to compare short-lasting cognitive stress with the one-hour test we applied.

The observed skin blood flow reduction during test is probably related to a gradually increasing sympathetic vasoconstrictor tone to skin arterioles and AV-shunts during cognitive stress [51]. However, we did not find any differences in finger BF development during the test between the three groups. This is in accordance with previous studies that have utilized finger temperature and pulse amplitude as indirect measures of finger blood flow during short-duration stress with generally negative results in TTH [25] and migraine [19].

We did find a delayed finger BF recovery profile *after *stress in TTH compared to controls and migraineurs. Another study has previously reported prolonged skin vasoconstriction in TTH (earlobe pulse volume and finger temperature) [29], which is in accordance with our findings. In addition, TTH patients had delayed pain recovery (Table 1) and delayed EMG recovery in the trapezius area [34]. Our findings in general fit well with the theoretical models of Eriksen & Ursin [1] and McEwen [2]. Our lack of HR adaptation in TTH is in accordance with McEwens concept of "allostatic load" which causes lack of adaptation to stress. Furthermore, the lack of skin BF recovery in TTH fits well both with the concept of "sustained arousal" in the model of Eriksen & Ursin, and with the concept of a prolonged response to a stressor in McEwens model.

The role of the autonomic nervous subsystems in TTH is not clear [25]. Because muscular blood flow in tender points is decreased in TTH [52], and because we observed increased skin vasoconstriction (reduced BF) during recovery after stress, which was correlated to low pain response during stress, it is possible that sympathetic dysregulation is involved, for instance as hyperactivity or hypersensitivity in the central autonomic network which again may be linked to increased central pain inhibition. It is also possible to explain this effect through pain-induced inhibition of sympathetic vasoconstriction in the skin however [53].

Recently, decreased muscle blood flow during muscle exercise was found in fibromyalgia patients, suggesting that muscle ischemia contributes to pain in these patients [54]. However, we were not able to measure intramuscular blood flow in the present study. Muscle blood flow is regulated differently from skin blood flow [55] and the direct relevance of observed skin blood flow changes to the relationship between muscle blood flow and pain perceived as muscular is accordingly uncertain.

Also in migraine, there are still many uncertainties about the role of autonomic nervous subsystems [17,19,24,56,57]. Some studies report autonomic dysfunction in migraineurs, such as orthostatic hypotension, noradrenergic or adrenergic hypofunction etc. [58-63], but not all studies report such autonomic dysfunction [64-66]. Many past studies have used procedures such as deep breathing tests, orthostatic tests, cold pressor tests and isometric work tests (sustained handgrip) and these responses are not directly comparable with autonomic response to cognitive stress of long duration used in the present study.

Cephalic and intracranial vessels may be regulated differently from peripheral vessels. Painful stimuli to tooth pulp induce a blood flow increase in orofacial areas [67]. In chronic TTH patients, previously published data indicate cranial vasodilatation [68]. In migraine, cephalic pulse amplitude may increase during a mental task in migraine [18] but results are not consistent across studies [19], and both deficient and normal vasoactivity has generally been reported in migraine [66]. Our results support the view that dysfunctional peripheral blood flow regulation is not a substantial part of migraine pathophysiology.

Although we did not measure perceived stress in this study, we believe that the measured perceived tension is an indirect measure of the level of stress. The Norwegian word "anspenthet" describe a feeling of general psychological and muscular tension perceived in stressful situations [69]. Tension responses did not differ, thus the level of stress seemed to be comparable between groups. However, TTH patients had a significantly less recovery from tension, indicating an inability to unwind after the stressor is removed [70].

As to what is perceived as stressful, TTH-patients may be more likely to appraise daily situations as stressful, with a tendency towards passive coping, compared to non-headache controls [25]. Because cognitive processing involving the prefrontal cortex can change the activity in the different parts of the periaqueductal grey matter (PAG), a difference in stress adaptive mechanisms may influence both the autonomic nervous system and pain control system in several ways, for instance by delaying sympathetic cardiovascular activation [71]. PAG is also important in pain control and in central sensitization, possibly explaining allodynia and hyperalgesia to pressure stimuli [72] and the increased stress-induced pain in TTH (Table 1, Figure 4).

## Conclusion

In conclusion, we report a possible lack of HR adaptation to stress in TTH patients, as well as a delayed finger skin BF recovery after stress and a correlation between finger skin BF recovery and the pain response. Also, TTH had an increase in SBP from the first 10 min to the last 10 min of the stress test, whereas controls and migraineurs did not. Autonomic responses to cognitive stress were not abnormal in migraine. We hypothesize that TTH patients have different stress adaptive mechanisms compared to controls and migraine patients, involving both cardiovascular activation and the pain control system. The motor system is also involved in responses to stress [73-75], and low-threshold motor unit activity may contribute to local metabolic changes and muscle pain [76,77]. However, because no associations between muscle activity and pain activation was found in a previous study [34], the present results suggest that cardiovascular responses are more closely linked to pain control than reflexes regulating muscle activity in TTH patients.

## Abbreviations

BF Blood flow

BP Blood pressure

DBP Diastolic blood pressure

EMG Electromyography

FB Feedback period

HR Heart rate

PAG Periaqueductal grey matter

SBP Systolic blood pressure

TTH Tension-type headache

UIR Uninstructed rest period

VAS Visual analogue scale

## Competing interests

The author(s) declare that they have no competing interests.

## Authors' contributions

RBL participated in acquiring data from the stress test, performed the statistical analyses and drafted the manuscript. TS participated in the design of the study, assisted in the statistical analyses and helped draft the manuscript. KBN participated in the design of the study, acquired data from the stress test and helped draft the manuscript. RHW participated in the design of the study. LJS participated in the design of the study and helped draft the manuscript. All authors read and approved the final manuscript.

## Pre-publication history

The pre-publication history for this paper can be accessed here:


